# Evaluation of the ex vivo liver viability using a nuclear magnetic resonance relaxation time-based assay in a porcine machine perfusion model

**DOI:** 10.1038/s41598-021-83202-3

**Published:** 2021-02-18

**Authors:** Qing OuYang, Guohai Liang, Xiaoyu Tan, Xiran He, Lin Zhang, Weijian Kuang, Jianxiong Chen, Shaoping Wang, Mingju Liang, Feng Huo

**Affiliations:** 1 Department of Hepatobiliary Surgery and Liver Transplant Center, The General Hospital of Southern Theater, Guangzhou, China; 2 Guangdong Shunde Industry Design Institute (Guangdong Shunde Innovative Design Institute), Shunde, Guangdong China; 3 Guangdong Devocean Medical Instrument Co., Ltd., Shunde, Guangdong China; 4 The MOE Key Laboratory of Laser Life Science, South China Normal University, Guangzhou, China

**Keywords:** Hepatology, Predictive markers

## Abstract

There is a dearth of effective parameters for selecting potentially transplantable liver grafts from expanded-criteria donors. In this study, we used a nuclear magnetic resonance (NMR) relaxation analyzer-based assay to assess the viability of ex vivo livers obtained via porcine donation after circulatory death (DCD). Ex situ normothermic machine perfusion (NMP) was utilized as a platform for viability test of porcine DCD donor livers. A liver-targeted contrast agent, gadolinium ethoxybenzyl diethylenetriamine pentaacetic acid (Gd-EOB-DTPA), was injected into the perfusate during NMP, and the dynamic biliary excretion of the Gd-EOB-DTPA was monitored by measuring the longitudinal relaxation time (T1). The longitudinal relaxation rate (R1) of the bile was served as a parameter. The delay of increase in biliary R1 during early stage of NMP indicated the impaired function of liver grafts in both warm and cold ischemia injury, which was correlated with the change of alanine aminotransferase. The preservative superiority in cold ischemia of dual hypothermic oxygenated machine perfusion could also be verified by assessing biliary R1 and other biochemical parameters. This study allows for the dynamic assessment of the viability of porcine DCD donor livers by combined usage of ex situ NMP and NMR relaxation time based assay, which lays a foundation for further clinical application.

## Introduction

The shortage of donor livers is a worldwide problem^1–3^. The use of expanded-criteria donor (ECD) livers meets the demands of patients on the waiting list for liver transplantation^4,5^. However, these livers are obtained from elder donors, steatotic donors, or circulated death donors (DCD), and these livers are more sensitive to cold ischemia injury in static cold storage (SCS) and have a higher risk of developing postoperative complications such as primary graft dysfunction (PGD), post-transplant cholangiopathy, or nonanastomotic biliary strictures than other livers are ^3,6,7^. Hence, defining proper index and criteria for testing the viability of ECD livers to identify potentially transplantable organs is important.

Ex situ normothermic machine perfusion (NMP) provides a practical platform for viability tests. This device maintains a liver temperature of 37 °C during preservation^8^. Researchers are now focusing on determining the index and criteria of viability tests for selection of transplantable organs^9,10^. Currently, the criteria for the selection of suitable donor livers during NMP make use of biochemical parameters including lactate clearance, transaminase concentration, and biliary pH or bicarbonate^4,5,9^. However, liver functional reserve including the uptake, metabolism and excretion could not be accurately reflected by these biochemical parameters alone.

Currently, gadolinium ethoxybenzyl diethylenetriamine pentaacetic acid (Gd-EOB-DTPA) is used in assessment of liver lesions and liver functional reserve in vivo^11^. Gd-EOB-DTPA can provide enhanced magnetic resonance imaging (MRI) phase information using MRI non-specific gadolinium contrast^12^. For normal livers, approximately 50% of the Gd-EOB-DTPA was incorporated by hepatocytes and finally excreted into the bile 20 min after the administration of one bolus^12–14^. In this way, quantitative comparison of the signal intensity measured by T1 relaxation time (T1) before and after contrast enhancement within a certain time could indicate the functional reserve. Decreased liver function may delay the development of bile duct images using Gd-EOB-DTPA. Previous studies have shown the feasibility of using Gd-EOB-DTPA-enhanced MRI for predicting liver function by measuring liver parenchymal or biliary tract enhancement on hepatobiliary-phase (HBP) MRI for patients^13,15^. However, these methods are now used in liver imaging in situ, but not ex situ. Furthermore, the device for NMP cannot be placed in the MRI apparatus for imaging because it contains iron-based material.

The main objective of this study was to assess the ex vivo liver viability by using a nuclear magnetic resonance (NMR) relaxation time based assay in a porcine machine perfusion model.

## Material and methods

### Study design and animal model

Bama miniature pigs weighed 45.2–53.7 kg (Liver weight = 1.184 ± 0.243 kg) were used for this study. The study was approved by the Clinic's Institutional Animal Care and Use Committee in General hospital of south theatre command in China (authorized number: 2019022501). All procedures performed in accordance with its guidelines.

### Donor procedure

Zoletil 50 (2–3.5 mg/kg) was injected intramuscularly to induce anesthesia. Endotracheal intubation was provided after intramuscular injection of atropine (0.02 mg/kg). Tramadol Hydrochloride (2 mg/kg, i.v.) was administered for analgesia. General anesthesia was then induced with propofol (mg/kg*h, i.v.) by means of a 21-gauge butterfly cannula inserted into an external marginal ear vein. Maintenance of anesthesia was induced with propofol (4–6 mg/kg), sevoflurane 2–3% and cis-atracurium (0.05 mg/kg). Ventilation mode was set as follows: VT: 8 ml/kg; RR: 16/min.

Donor animals underwent a midline laparotomy. The cystic duct was ligated. The bile duct was dissected and the bile was allowed to drain freely. The major vessels in hepatic hilus were dissected. The aorta and inferior vena cava were cannulated with a large cannula. Pigs received heparin at 500 IU/kg of body weight 5mins prior to cross-clamping, then blood was quickly drawn from the aorta and inferior vena cava. The blood was collected in acid citrate dextrose bags for later NMP (approximate 1200–1500 ml). After blood collection, cardiac arrest by intra-cardiac infusion of potassium chloride (20 mEq) was provided. The donor would remain untouched for a period to simulate the procedure of obtaining DCD donor. 0, 30 or 60 min after the cardiac arrest were recorded as the warm ischemia time (WIT). In order to study the effect of different degrees of warm ischemia on liver function, the grafts were divided into three groups according to the warm ischemia time (WIT: 0′, n = 5; 30′, n = 6; 60′, n = 6). The donor liver was perfused with 2 L of University of Wisconsin cold storage solution (UW-CS) both portally and arterially. Liver grafts were quickly removed with standard technique and preserved in UW-CS with ices. The grafts were prepared for cannulating. Then the donor hepatic artery, portal vein, and bile duct were cannulated. The liver was connected to a prototype of ex situ normothermic machine perfusion device.

### NMP

In brief, the device consisted of a container for the liver, two centrifugal pump revolutions (Sorin centrifugantion pump revolution, Germany) delivering continuous flow to the portal vein and pulsatile flow to the hepatic artery; Two membrane oxygenator (WEGO, China) and a measurement and control device connected to an interface. The catheter is made by medical PVC and silica gel and could detect the blood pressure and blood flow by sensors. The pulsatile pump delivered perfusate from the container, through the oxygenator, and into the hepatic artery; the continuous pump perfused the portal vein also with oxygenation (Supplementary Fig. 1A,B). Oxygen flow was constantly to both portal vein and hepatic artery and the FiO_2_ was 60%. Before placing the liver, the machine was primed with 2 L of whole blood mixed with a machine perfusion solution. The composition of machine perfusate solution was list in Supplementary Table 1. Arterial perfusion pressures were maintained at 80/60 mmHg (systolic pressure/diastolic pressure) (Supplementary Fig. 1D–E) and the portal vein was perfused with a constant flow with 0.5 ml/min/g (liver weight) at the first hour, and elevate to 0.75 ml/min/g (liver weight). The portal vein related hemodynamic data were shown in supplementary Fig. 1A–C.

**Figure 1:**
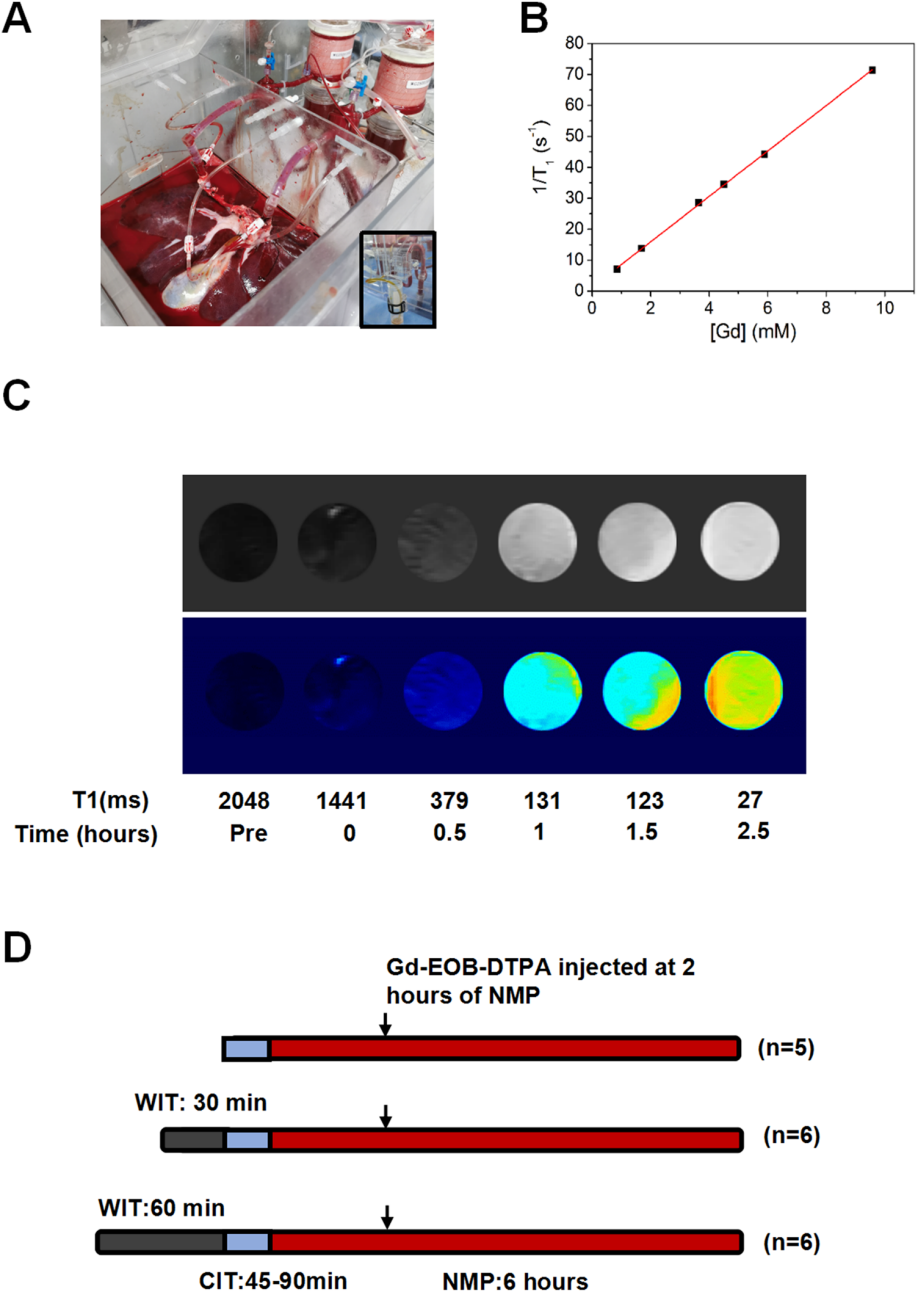
Dynamic change of biliary R1 correlates significantly with the concentration of Gd in bile during NMP. (**A**) Representative image of a liver graft in the NMP device. Bile samples were collected dynamically. (**B**) After Gd-EOB-DTPA was injected in the circulated perfusate 2 h after initiation of NMP, six bile samples were collected at different time points. The T1 relaxation time of the bile samples were measured using an NMR relaxation analyzer. Gd concentration in corresponding samples was analyzed via inductively coupled plasma-atomic emission spectrometry (ICP-AES). Significant linear correlation between Gd concentration and biliary relaxation rate (R1 = 1/T1) is shown (residual sum of squares = 0.73925; Adj. R-square = 0.99965; 1/T1 Intercept: 1.30671, slope: 7.3405). (**C**) T1-weighted MR images of the bile samples. Corresponding T1 value and the time point for sample collection after Gd-EOB-DTPA injection of each bile sample are listed below. (**D**) The basic model for viability test of liver grafts during NMP. Livers were obtained after draining the blood. Following 0 min, 30 min and 60 min of warm ischemia and 45–90 min of cold ischemia (preparing process), livers were allowed to cannulate to NMP and divided into 3 groups (WIT:0′, n = 5; WIT:30′, n = 6; WIT:60′, n = 6). Gd-EOB-DTPA was injected 2 h after starting NMP, and bile samples were collected for R1 measurement.

### SCS and DHOPE

Donor grafts were preserved at 4 °C with UW-CS. Cold ischemia time was recorded from grafts preparing period to SCS period. Then to study the advantage of DHOPE in preservation at 4 °C, the liver grafts were connected to the machine with continuous perfusion at 4 °C. Oxygen flow was provided 1000 mL/min constantly. Arterial perfusion pressure was maintained at 25 mmHg and the portal vein was perfused with a constant flow with 200 ml/min. Before placing the liver, the machine was primed with 2 L of UW-MP. The temperature in the perfusate was detected and controlled at 4 °C by a semiconductor system. Flows and pressure in artery and portal vein were recorded.

### Dynamic test for biliary longitudinal relaxation time

The commercial name of the contrast agent of Gd-EOB-DTPA we used in the experiment was Primovist. And the dosage of the agent was 3 ml/kg (liver weight). After 2 h of NMP, Gd-EOB-DTPA was injected into the perfusate. Firstly, bile samples were collected at 30 min intervals after the injection, and the longitudinal relaxation time was detected by a 1.0 T MRI. 2 mL of the secreted bile was collected at each time point and centrifuged at 2000 rpm for 2 min. The supernatant was placed in a 0.2 mL centrifuge tube for T1 relaxation time measurement. A 0.5 T NMR analyzer (MINIPQ001, Shanghai Niumag Co., China) was used for the measurement. T1 was acquired via an inversion-recovery (IR) pulse sequence with a TR of 9000 ms, TE of 14 ms, and 20 inversion recovery points ranging between 10 and 5000 ms. R1 (R1 = 1/T1) was used as the parameter for the viability test. All measurements were carried out in triplicate and data were expressed as mean ± SD.

Following relaxation time measurement, T1-weighted MR images of the samples were acquired on a 1.0 T MRI (NM-G1, Shanghai Niumag Co., China) using a routine spin echo (SE) sequence. The parameters were as follow: 32 °C, TR/TE = 40 ms/16.5 ms, NS = 4, field of view (FOV) = 40 × 40 mm^2^, slice thickness = 1.5 mm, matrix = 192 × 256.

### Blood and biliary viability test

At the beginning of laparotomy, we isolated the bile duct and connect with a catheter. Blood and bile were sampled in the donor pigs as a baseline and during NMP hourly. Serum levels of transaminase, lactate, blood glucose and biliary pH were measured.

### Histological analysis

Biopsies of the liver were collected at 6 h after NMP. All biopsies were fixed in formalin and paraffin-embedded. Slides were stained with hematoxylin and eosin (H&E) and assessed by light microscopy. Common bile duct was collected at 2 h after NMP. Evaluation of BDI was assessed by using an established, clinically relevant, histological BDI grading system^17^ as described by Supplementary Table 2. The presence of necrosis in bile duct epithelium, the peribiliary glands loss, blood congestion and infiltration of inflammatory cells in bile duct biopsies have been observed and recorded.

### Transmission electron microscopy (TEM)

The fixed procedure was as described^16^. Samples were immersed with 2% paraformaldehyde and was further fixed with sequential incubation with 1 and 2% glutaraldehyde in PBS at 4 °C for 24 h. Post-fixed procedure was provided with 1% osmium tetroxide, electron-stained with 3% uranyl acetate, and embedded in an Epon-Araldite mixture. Then ultrathin sections were cut with approximately 60 nm by a ultrathin microtome (Leica EM UC7) and detected by a Hitachi H-7700 electron microscope (Hitachi, Tokyo, Japan).

### Data and statistical analysis

Data was analyzed with the SPSS 22 statistical package (IBM, Chicago, IL, USA). Two-way ANOVA was used for the analysis of differences among three groups (WIT: 0′, 30′, 60′). Bonferroni post-test was used for analysis the differences between two groups at every time-points during NMP. Two-way ANOVA plus Bonferroni post-test was also used for analyzing the DHOPE group and SCS group at every time-point during NMP. Correlations were calculated using the Spearman correlation test. Data was presented as mean ± SD (standard deviation) and was considered significant at the level of *p* < 0.05.

## Results

### Detection of biliary longitudinal relaxation time after Gd-EOB-DTPA injection during NMP

The NMP device was utilized for the maintaining of liver grafts at 37℃ (Fig. 1A). After 2 h of NMP, Gd-EOB-DTPA was injected into the perfusate. Bile samples were collected at 30 min intervals after the injection, and the longitudinal relaxation time was detected by a 1.0 T MRI. longitudinal relaxation rate R1 = 1/T1 [s^−1^] of the samples was calculated. Before the Gd-EOB-DTPA injection, the bile showed a small background T1. Once Gd-EOB-DTPA was injected into the perfusate, the change in biliary T1 appeared 30 min after Gd-EOB-DTPA injection (Fig. 1C). To visually depict the change in T1 of the samples, T1-weighted MR images of the cross-section of six samples were acquired following the measurement of T1 (Fig. 1C). Next, to verify the relationship between T1 and Gd excretion in bile, six bile samples from different animals were collected and the contained Gd concentration was analyzed using inductively coupled plasma-atomic emission spectrometry (ICP-AES). An excellent linear correlation between Gd concentration and biliary 1/T1 (R1) was observed (Fig. 1B). Therefore, we used biliary R1 as a new indicator of viability, as its change directly reflected the excretion of Gd-EOB-DTPA.

### Change of biliary R1 reflects the injury of hepatocyte caused by warm ischemia

**Figure 2:**
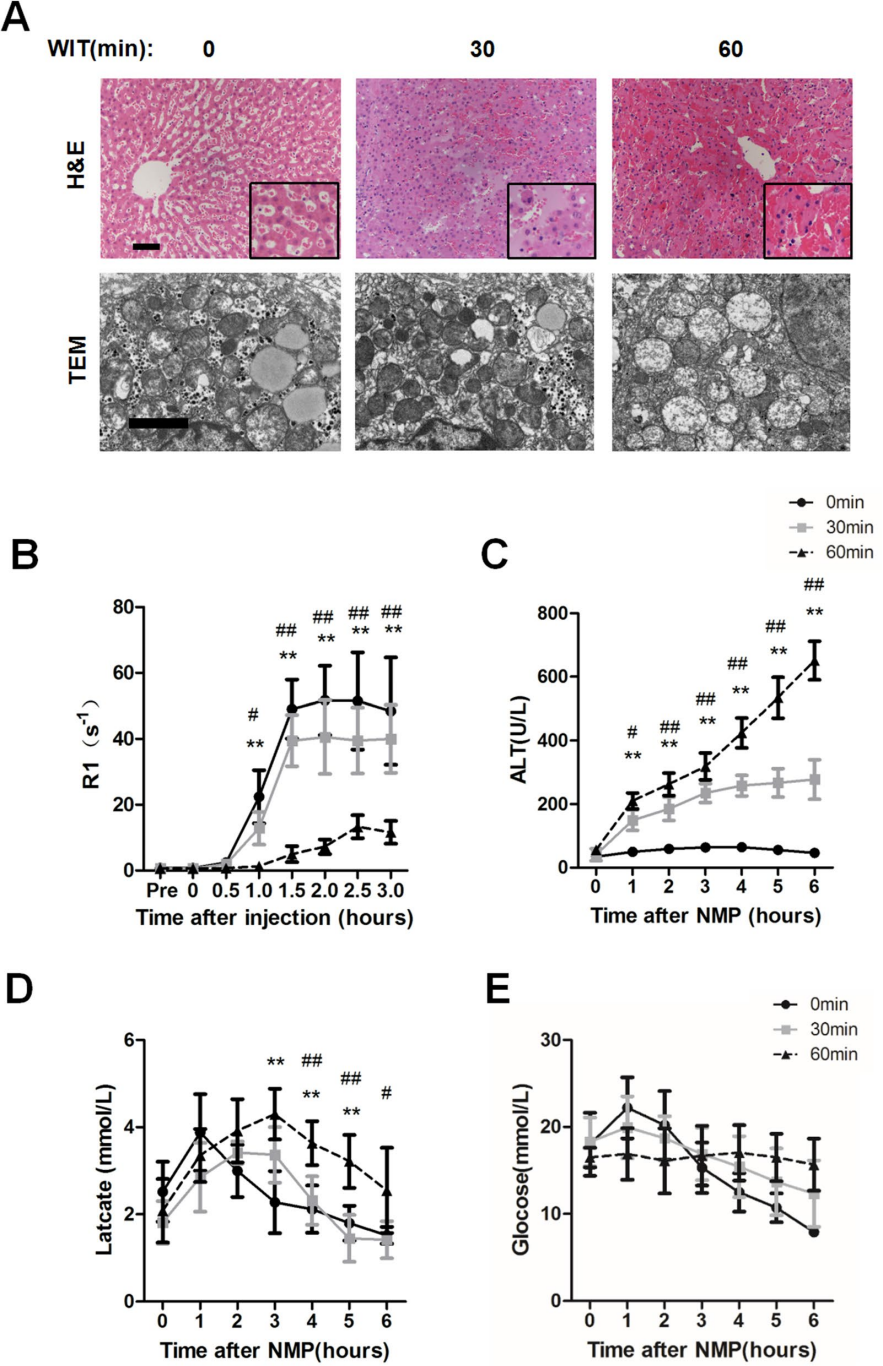
Detection of biliary R1 and biochemical parameters in DCD livers with different WIT during NMP. (**A**) Histological (top) and transmission electron microscopy (TEM) (bottom) images of hepatocytes were obtained from the indicated groups 6 h after NMP initiation. The scale bars in the histological and TEM images represent 100 μm and 2 μm, respectively. (**B**–**E**) Evaluation of biliary R1, ALT, perfusate lactate, and perfusate glucose levels of grafts receiving different warm ischemia time. Grafts were divided into three groups according to the warm ischemia time (a. 0′; b. 30′; c. 60′). After cannulating to identical NMP, bile and blood samples were collected at the indicated time points (data represent mean ± SD. Two-way ANOVA was used for the analysis of differences among three groups. Group a (0′) vs. Group c (60′): *P < 0.05, **P < 0.01; Group b (30′) vs. Group c (60′): #*P* < 0.05, ##*P* < 0.01).

In order to study the effect of warm ischemia on liver function, the grafts were divided into three groups according to the warm ischemia time (WIT: 0′, n = 5; 30′, n = 6; 60′, n = 6). (Fig. 1D). Liver biopsy was provided after 6 h of NMP. The H&E staining of liver grafts that endured 0′, 30′ and 60′ of warm ischemia revealed that little difference in hepatocyte was found among three groups (Fig. 2A). However, transmission electron microscopy (TEM) images of these samples indicated that mitochondrial swelling and disappearance of cristae in hepatocytes were observed in grafts endured 60′ of warm ischemia, but were not observed in hepatocytes that endured 0′ and 30′ of warm ischemia (Fig. 2A).

Next, biliary R1 after injection of Gd-EOB-DTPA was detected at 30 min of intervals during NMP. Bile was collected before and after Gd-EOB-DTPA injection at indicated time points for measurement of R1. A rise in R1 means that a strong contrast signal could be detected in the bile. Then, the biliary R1 increased in the groups with WIT for 0′ and 30′ after 1 h after injection of Gd-EOB-DTPA. However, the biliary R1 was significantly lower in the group of WIT for 60′ than that in the other two groups at 1hour (60′) post-injection (biliary R1 at 60′ post-injection: 0′ group *vs.* 30′ group *vs.* 60′ group: 22.4 ± 8.06 s^−1^ *vs.*12.9 ± 4.96 s^−1^ *vs.*1.3 ± 0.37 s^−1^, *P* < 0.01) (Fig. 2B).

Other conventional parameters for viability test, such as alanine aminotransferase (ALT), perfusate lactate, and perfusate glucose levels were also assessed hourly during NMP (Fig. 2C–E). ALT level could vary significantly among the three groups after reperfusion (*p* < 0.01). ALT level was significantly higher in the group of WIT for 60′ after NMP (Fig. 2C). Perfusate lactate level were decreased in all three groups. Perfusate lactate level started reducing in group of WIT for 60′ at 4 h after NMP , but still higher than groups of WIT for 0′ and 30′ group (perfusate lactate at 4 h after NMP: WIT 0′ group *vs.* 30′ group *vs.* 60′ group: 2.12 ± 1.04 mmol/L *vs.* 2.31 ± 0.56 *vs.* 3.63 ± 0.50 mmol/L, *P* < 0.01) (Fig. 2D). There was no significant difference in perfusate glucose level among three groups (Fig. 2E). Correlation analysis was conducted between biliary R1 (60′) and conventional parameters at 4 h after NMP. As expected, biliary R1 (60′) and ALT level at 4 h after NMP were strongly correlated (Fig. 4A). But significant correlation was not found in biliary R1 (60′) and perfusate lactate at 4 h after NMP (Fig. 4B). These results suggested that the delay of increasement of biliary R1 at early stage of NMP may reflect the impaired function of liver grafts.

### Detection of biliary duct injury (BDI) in warm ischemia injury

**Figure 3:**
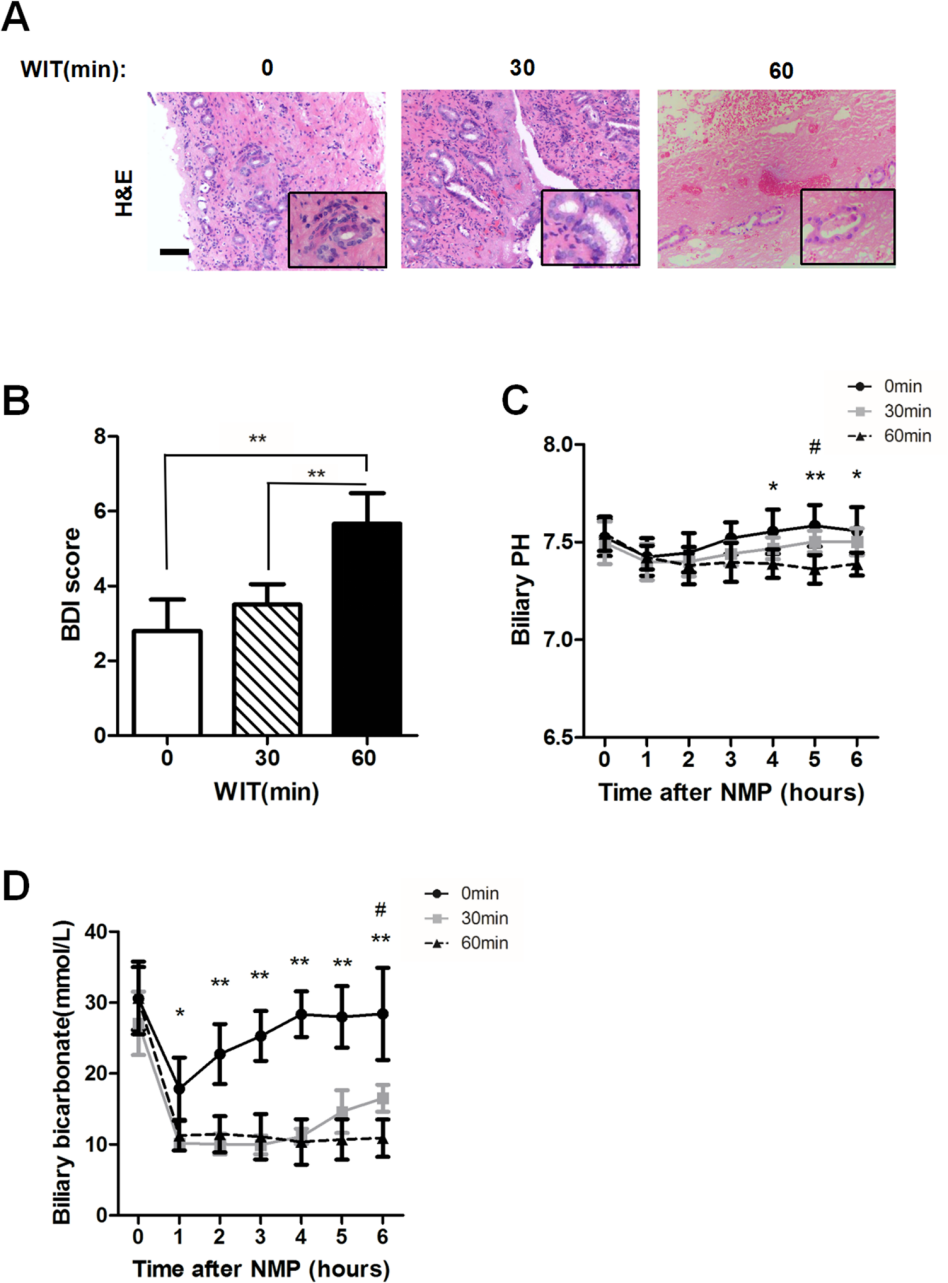
Detection of biliary duct injury (BDI) in different degree of warm ischemia injury. (**A**) Histological images of the bile duct with indicated WIT (0′, 30′, 60′). Scale bar = 100 μm. (**B**) Evaluation of BDI score of the bile duct with indicated WIT. (data represent mean ± SD. Student’ s t-test was used for analyzing between groups.*P < 0.05, **P < 0.01). (**C**) The evaluation of biliary pH of livers with different WIT (0′, 30′, 60′). Bile samples were collected at indicated time points after NMP. Biliary pH was tested at different time points after NMP initiation (data represent mean ± SD. Two-way ANOVA was used for analyzing the differences among three groups. Group a (0′) vs. Group c(60′): *P < 0.05, **P < 0.01;Group b (30′) vs. Group c (60′): #*P* < 0.05, ##*P* < 0.01). (**D**) The evaluation of biliary bicarbonate of livers with different WIT (0′, 30′, 60′). Bile samples were collected at indicated time points after NMP. Biliary bicarbonate was tested at different time points after NMP initiation (data represent mean ± SD. Two-way ANOVA was used for analyzing the differences among three groups. Group a (0′) vs. Group c (60′): *P < 0.05, **P < 0.01; Group b (30′) vs. Group c (60′):#*P* < 0.05, ##*P* < 0.01).

Biliary duct injury (BDI) is also an important factor of functional evaluation for donor livers. Recently, Porte reported that the degree of BDI can be assessed via histological scoring^17^. Herein, we employed these histological items to evaluate BDI from the histological samples of the bile duct 2 h after initiation of NMP. The representative H & E staining of bile ducts in three groups were shown in Fig. 3A. The mean BDI score of three groups were shown in Fig. 3B. Severe change after ischemia and reperfusion injury were observed in liver grafts with WIT for 60′. As expected, the BDI score in group with WIT for 60′ was significantly higher than the other two groups. (BDI score: WIT 0′ group *vs.* 30′ group *vs.* 60′ group: 2.8 ± 0.84 *vs.* 3.50 ± 0.55 *vs.* 5.67 ± 0.82, *P* < 0.01).

**Figure 4:**
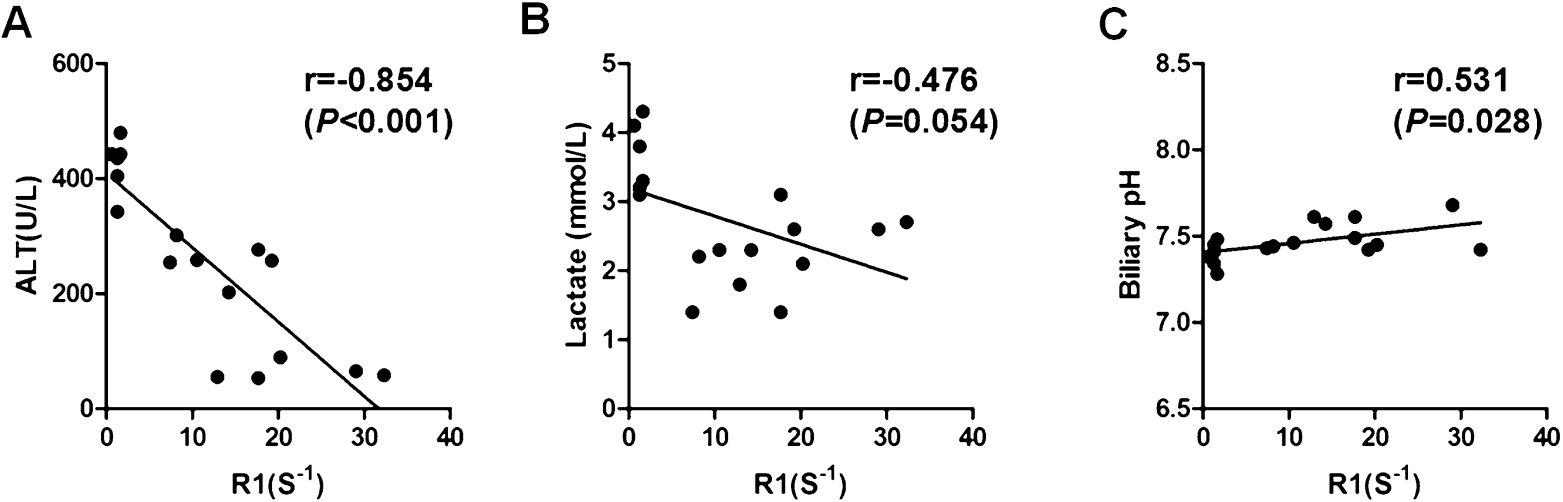
Correlation analysis between biliary R1 at early stage and biochemical parameters at 4 h of NMP. Correlation analysis was conducted between biliary R1 at 1 h after Gd-EOB-DTPA injection (60′) and conventional biochemical indexes at 4 h after NMP (ALT, perfusate lactate and biliary pH). (**A**) ALT value at 4 h after NMP had a strong correlation with values of biliary R1 (60′) (Spearman; r, − 0.854; *p* < 0.001). (**B**) Lactate value at 4 h after NMP had no significant correlation with values of biliary R1 (60′) (Spearman; r, − 0.476; *p* = 0.054). (**C**) There is a moderate between values of R1 (60′) and Biliary pH at 4 h after NMP (Spearman; r, 0.531; *p* = 0.028).

Parameters assessed from bile could also reflect the cholangiocyte function, such as biliary pH and bicarbonate. Here, biliary pH and bicarbonate were assessed at different time points for livers with different WIT. The results showed that biliary pH increased faster in livers with WIT for 0′. Biliary pH was significantly lower since 4 h after NMP in livers with WIT for 60′ (Biliary pH at 4 h of NMP: WIT 0′ group vs. 30′ group vs. 60′ group: 7.58 ± 0.11 vs. 7.50 ± 0.05 vs. 7.36 ± 0.07, *P* < 0.01) (Fig. 3C).The fast release of bicarbonate in bile was observed in livers with WIT for 0′. Biliary bicarbonate was significantly lower in livers with WIT for 60′ even after 6 h of NMP (Fig. 3D). (Biliary bicarbonate at 6 h of NMP: WIT 0′ group vs. 30′ group vs. 60′ group: 28.42 ± 6.51 vs. 16.52 ± 1.90 vs. 10.88 ± 2.63, *P* < 0.01) Correlation analysis was also conducted between biliary R1 at 1 h after Gd-EOB-DTPA injection (60′) and biliary pH level at 4 h after NMP. However, there was a moderate correlation between values of R1 (60′) and Biliary pH at 4 h after NMP (Fig. 4C).

### Detection of biliary R1 in liver grafts treated with DHOPE after circulatory death donation

**Figure 5:**
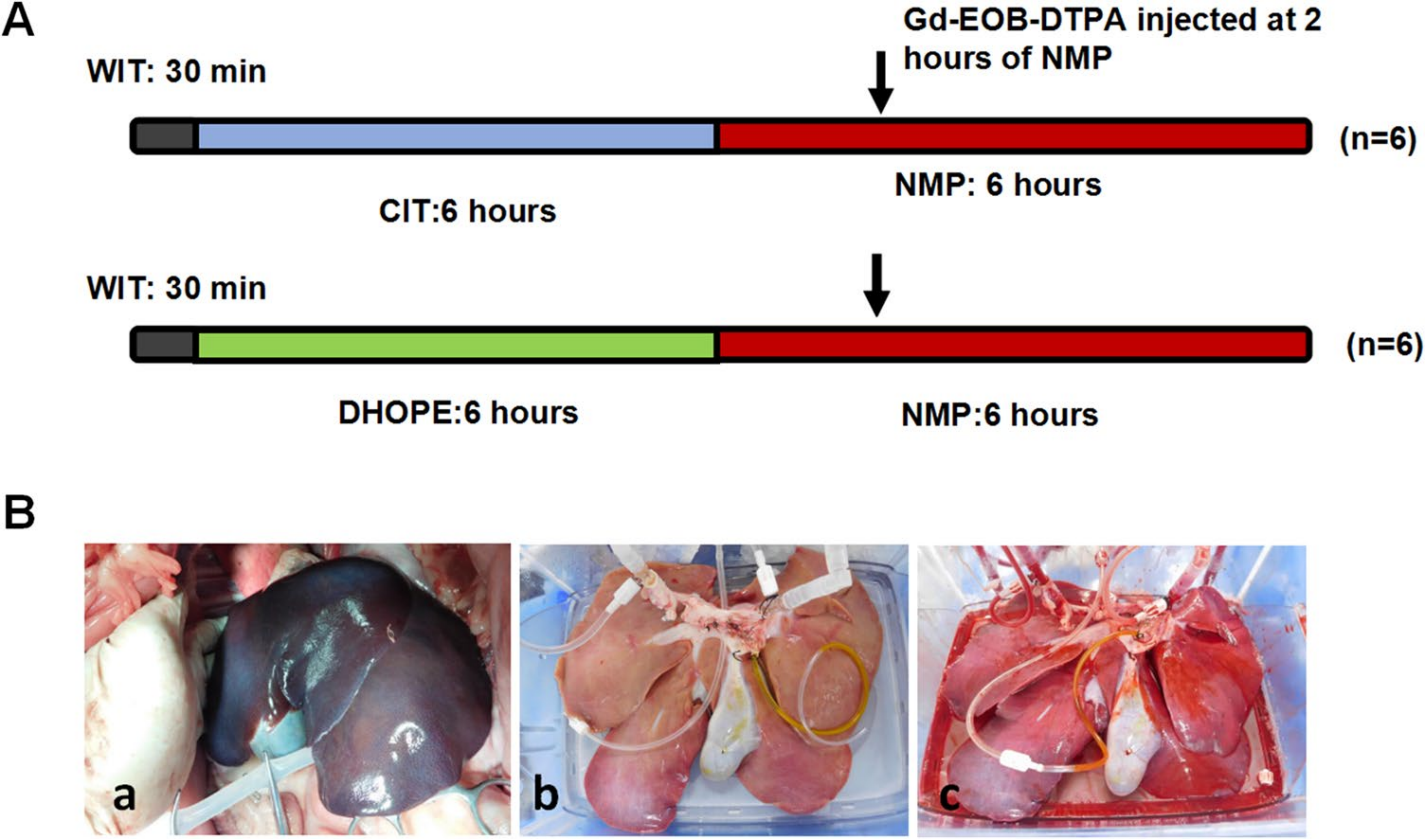
Models of viability test on liver grafts receiving SCS or HOPE treatments. (**A**) Livers were preserved in static cold storage (n = 6) or HOPE (n = 6) for 6 h after 30 min of warm ischemia. Then livers were allowed to cannulate to NMP. Gd-EOB-DTPA was injected 2 h after starting NMP, and bile or blood samples were collected at indicated time points. (**B**) Representative images of the liver treated with warm ischemia in situ (a), liver graft connected to the ex situ DHOPE in 4 °C (b), and liver graft connected to the ex situ NMP (c).

**Figure 6:**
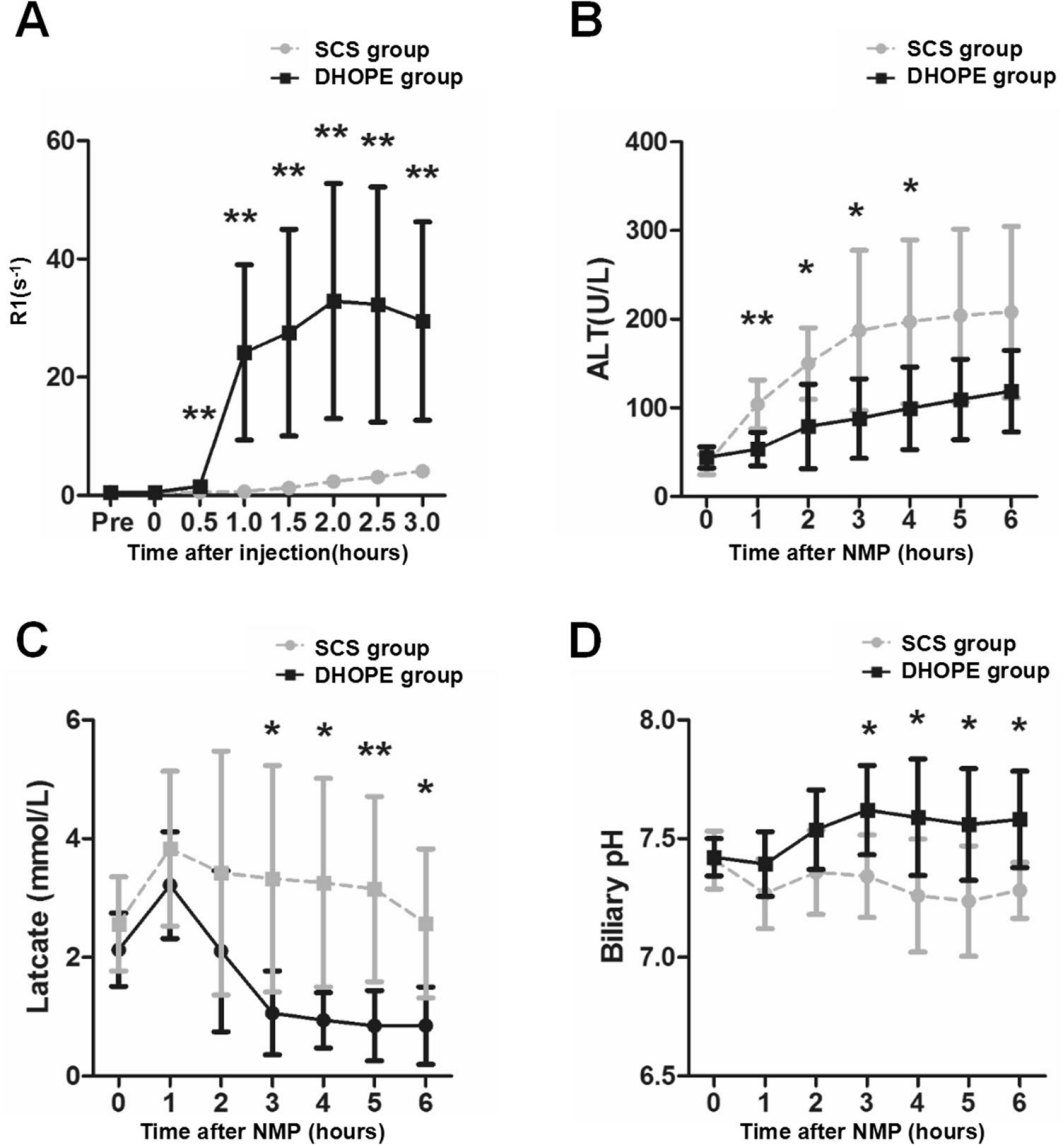
Evaluation of biliary R1, ALT and lactate levels, and biliary pH during NMP after 6 h of DHOPE or SCS treatments. (**A**–**E**) Analysis of biliary R1, ALT, perfusate lactate, and biliary pH during NMP following 6 h of DHOPE or SCS treatment at the indicated time points. Data represent mean ± SD. Two-way ANOVA was used for analysis the differences between two groups. DHOPE group vs. SCS group: *P < 0.05, **P < 0.01).

In order to study the effect of combined ischemia injury (warm ischemia and cold ischemia) on grafts quality, long term of static cold storage (SCS) was added in our model. As shown in Fig. 5A, after 30 min of warm ischemia, liver graft was preserved in SCS for 6 h before cannulating to the NMP device. The biliary R1 was tested at indicated time points. As expected, the level of biliary R1 was reduced in the SCS group during NMP and failed to increase even after 4 h of NMP (Fig. 6A).

DHOPE has been reported to be beneficial for donor preservation under cold ischemia condition. To confirm that, we compared the preservative effect of DHOPE (n = 6) with SCS (n = 6) by detecting parameters, including lactate, ALT, biliary pH, and biliary R1 in following NMP. The procedure was shown in Fig. 5A. Representative images of the liver treated with warm ischemia in situ (a), liver graft connected to the ex situ DHOPE in 4 °C (b), and liver graft connected to the ex situ NMP in 37 °C were showed in Fig. 5B.

A significant difference in dynamic biliary R1 was observed between the DHOPE and SCS groups (Fig. 6A). As shown above, a delayed of R1 increase was observed in the SCS group. In contrast, biliary R1 increased obviously 1 h after the injection of Gd-EOB-DTPA and peaked at 2 h in the DHOPE group, while it remained below 1 s^−1^ within 2 h after the injection of Gd-EOB-DTPA in the SCS group (R1 at 2 h post-injection: DHOPE *vs*. SCS: 32.89 ± 19.88 s^−1^ *vs*. 2.36 ± 0.67 s^−1^, *P* < 0.01). (Fig. 6A).

Dynamic changes in other parameters were also studied. The pattern of ALT alteration varied in the two groups. In the SCS group, ALT level increased obviously within the 3 h after reperfusion and still increased with time during NMP (ALT: 3 h after NMP: DHOPE *vs*. SCS:88.00 ± 44.85 U/L *vs*. 187.3 ± 90.52 U/L, *P* < 0.05), while in the DHOPE group, ALT increased moderately and remained below the average ALT value in the SCS group at the same time point during NMP (Fig. 6B). The perfusate lactate levels decreased in both groups 2 h after NMP. However, a significantly lower lactate level was observed in the DHOPE group during the 3 h in NMP (lactate value at 3 h: DHOPE *vs*. SCS: 1.06 ± 0.71 mmol/L *vs*. 3.31 ± 1.91 mmol/L, *P* < 0.05) (Fig. 6C). Biliary pH fluctuated in the two groups in the first two hours of NMP. In the DHOPE group, biliary pH increased faster and peaked at 4 h, while in the SCS group, biliary pH remained low during NMP (biliary pH at 4 h after NMP: DHOPE *vs*. SCS: 7.59 ± 0.25 *vs*. 7.26 ± 0.24, *P* < 0.05) (Fig. 6D).

## Discussion

Machine perfusion was developed to improve the quality of preservation for donor grafts^8,18–21^. Two major perfusion strategies, hypothermic and normothermic techniques, are currently used in clinical settings^1,6,22–24^. Normothermic perfusion allows the liver grafts to remain alive with normal metabolism, which provides a platform to determine the viability prior to transplantation^10,25^. Currently, detection of multiple biochemical parameters during NMP to predict different modes of potential graft failure were considered as the best means of assessing the viability of liver grafts^8,9^. However, conventional biochemical parameters as bilirubin, albumin, transaminase, perfusate lactate, perfusate glucose, and biliary pH could not directly or dynamically indicate the real-time metabolic state of the liver graft^15,26^. The test for functional reserve of liver grafts including uptake, metabolism, and excretion, should be considered in the viability test for ECD livers.

The indocyanine green (ICG) clearance test has been widely used as a quantitative indicator for evaluation of the liver functional reserve in vivo before hepatic surgery^27^. A similar mechanism (i.e., the organic anion transporter) is considered responsible for uptake of gadoxetate disodium and ICG in the liver. Thus, the excretion speed of Gd-EOB-DTPA could indicate the functional reserve in liver grafts with similar degrees of injury like ICG clearance^14,28^. However, conducting the ICG clearance test during NMP is complex and unstable owing to the method of assessing ICG fluorescence during ex situ NMP^29^. In contrast, the NMR-based assay reported in this study is suitable for complex samples such as bile because it is based on the magnetic properties of Gd-EOB-DTPA. The signal reflected by longitudinal relaxation time is closely associated with the dynamic excretion of the contrast agent. In this way, the entire test process for biliary R1 using NMR based assay is stable and fast.

The speed of Gd-EOB-DTPA excretion in bile, which is closely associated with the biliary R1 could reflect the real-time function of liver grafts. For porcine liver grafts with warm ischemia time of 0′ and 30′, a strong magnetic signal of Gd-EOB-DTPA could be detected in the bile sample at early stage after injection. Thus the presence of signal of contrast agent in bile after 30 min of injection of Gd-EOB-DTPA could be considered as an indicator of a good working state in the liver graft. Then, long periods of warm ischemia (60′) caused a visible delay in excretion of Gd-EOB-DTPA into the bile which was reflected by lower biliary R1. Besides, the correlation between ALT value at 4 h after NMP and Biliary R1 at 1 h after Gd-EOB-DTPA injection was observed (Fig. 4A). In the morphological study, swollen mitochondria without cristae were observed in the hepatocytes with WIT of 60′ in transmission electron microscopy (TEM). These results may indicate that the delay in Gd-EOB-DTPA excretion could predict impaired liver function in DCD liver grafts.

Many experts have confirmed that long term of cold ischemia could aggravate warm ischemia injury in DCD donor^30^. Biliary epithelial cells in the liver are sensitive to long term cold ischemia, which leads to dysfunctional regulation of ions and irreversible disruption of the plasma membrane^1,31^. So in this study, we hope to simulate some clinic scenarios through a combined ischemic injury model (30 min of warm ischemia plus 6 h of cold ischemia) in order to observe changes of biliary R1 and other traditional biochemical indicators. The preservation advantage of hypothermic oxygenated machine perfusion has already been confirmed^1,31,32^. Fondevila et al. showed that hypothermic oxygenated machine perfusion was more advantageous to preservation than cold storage for warm ischemia damaged livers^30^. Recently, a clinical study showed that the 5-year outcomes of DHOPE-treated DCD liver transplants were similar to those of DBD primary transplants and superior to those of untreated DCD liver transplants^33^. In our study, the preservative advantage of HOPE in 4 °C could also be reflected by the changes of biliary R1 as well as other conventional parameters. The increase in biliary R1 in the DHOPE group at early stage may indicate that the impaired liver function had been rescued by oxygenated perfusion.

Application of biliary R1 as an indicator for donor selection still needs further experimental verification. In a porcine model of DCD, grafts with 70 min of warm ischemia were successfully transplanted and had 100% survived^34^. This may indicate that the ECD livers with long term of ischemia time still have the potential for usage. Then, any single biochemical parameter could not generally reflect the viability of these ECD livers. Therefore, it is currently considered that the evaluation of viability of liver grafts could only be achieved by combining multiple index. Change of biliary R1 could reflect the ability of uptake, metabolism and excretion in liver graft, which could be a reliable complement to the proposed viability criteria. Transplant experiments to verify the correlation between biliary R1 and graft survival is indispensable, which will be carried out in future experiments. As Gd-EOB-DTPA and the NMP are currently utilized as clinical approaches, the clinic verification tests and the criteria set will be performed soon.

In conclusion, we demonstrated the feasibility of detecting ex vivo liver viability with combination of gadolinium-enhanced MRI and NMP device, as it is a fast technique based on already existing devices developed for the clinics^35,36^. Transplant experiments to verify the correlation between biliary R1 and graft survival is indispensable, which will be carried out in future.

## Supplementary Information

Supplementary Figure S1.

Supplementary Legends.

Supplementary Table S1.

Supplementary Table S2.
