# Supplementary figures and images for: Evaluation of the ex vivo liver viability using a nuclear magnetic resonance relaxation time-based assay in a porcine machine perfusion model
Source: Sci Rep. 2021 Feb 18;11:4117. doi: 10.1038/s41598-021-83202-3 (PMC7892848; doi:10.1038/s41598-021-83202-3)

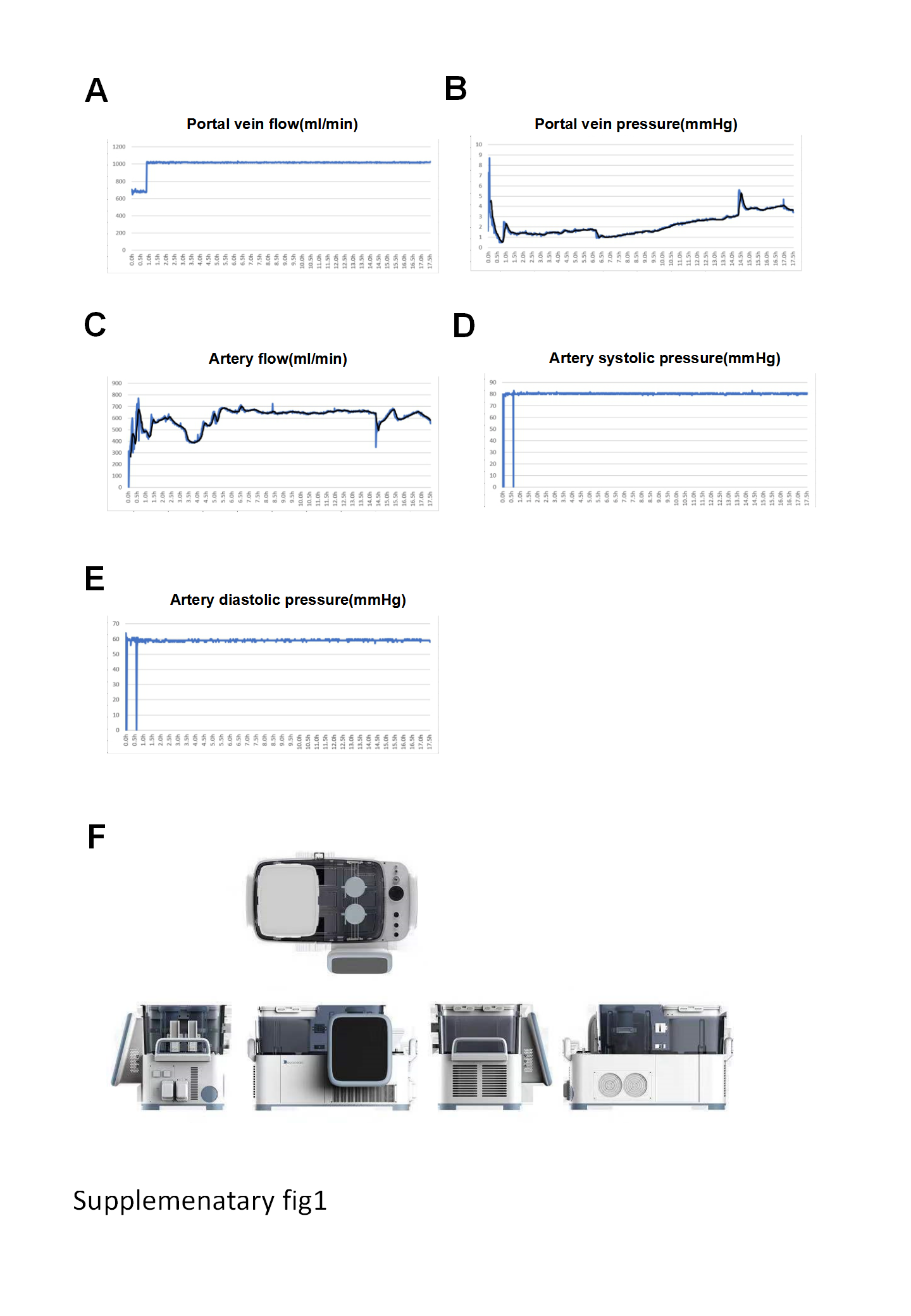

Supplement: Supplementary file 1 — Supplementary Figure S1. [file 41598_2021_83202_MOESM1_ESM.tif]
