# Supplementary material for: Evaluation of the ex vivo liver viability using a nuclear magnetic resonance relaxation time-based assay in a porcine machine perfusion model
Source: Sci Rep. 2021 Feb 18;11:4117. doi: 10.1038/s41598-021-83202-3 (PMC7892848; doi:10.1038/s41598-021-83202-3)
Supplement: Supplementary file 2 — Supplementary Legends. [file 41598_2021_83202_MOESM2_ESM.docx]

**Supplementary information**

**Evaluation of the *Ex-Vivo* Liver Viability Using a Nuclear Magnetic Resonance Relaxation Time-based Assay in a [Porcine Machine Perfusion Model](http://www.researchgate.net/publication/306084827_Steroids_can_reduce_warm_ischemic_reperfusion_injury_in_a_porcine_DCD_model_with_EVLP_evaluation)**

Qing OuYang^1^*, Guohai Liang^4^*,Xiaoyu Tan^1^*, Xiran He^2^, Lin Zhang^3^, Weijian Kuang^2^, Jianxiong Chen^1^, Shaoping Wang^1^, Mingju Liang^2#^, Feng Huo^1#^

^1^Department of Hepatobiliary surgery and liver transplant center, The General Hospital of South Theater, Guangzhou, China.

^2^Guangdong Shunde Industry Design Institute (Guangdong Shunde Innovative Design Institute), Shunde, Guangdong, China.

^3^Guangdong Devocean Medical Instrument Co.,Ltd. Shunde, Guangdong, China.

^4^The MOE Key Laboratory of Laser Life Science, South China Normal University, Guangzhou, China.

*These authors contributed equally to this work.

^#^Corresponding authors.

**Correspondence**

Feng Huo

[gzhuofeng@163.com](mailto:gzhuofeng@163.com)

Mingju Liang

391129243@qq.com

**Supplementary Fig. 1** Hemodynamic parameter setting and recording during NMP

1. B). Hepatic artery related hemodynamic data was recorded by the device; (D-E). Portal vein related hemodynamic data was recorded by the device. (F). Machine perfusion equipment appearance design.

**Supplementary table 1.** Composition of perfusate

**Supplementary table 2.** Items and degree of bile duct injury in liver grafts of this study accessed via histological scoring
