# Supplementary material for: Evaluation of the ex vivo liver viability using a nuclear magnetic resonance relaxation time-based assay in a porcine machine perfusion model
Source: Sci Rep. 2021 Feb 18;11:4117. doi: 10.1038/s41598-021-83202-3 (PMC7892848; doi:10.1038/s41598-021-83202-3)
Supplement: Supplementary file 3 — Supplementary Table S1. [file 41598_2021_83202_MOESM3_ESM.docx]

| 5% Human serum albumin | 100-150ml |
| --- | --- |
| Whole blood | 1.2-1.5L |
| 2.5% NaHCO_3_ | 21ml |
| 10% CaCL_2_ | 7ml |
| Heparin | 5000U |
| Cefoxltin | 1g |
| Metronidazole | 500mg |
| Sodium taurocholate | 5g |
| Short acting insulin | 72U |
| Total parenteral nutrition solution | 250-500ml |

**Supplementary table 1.** Composition of perfusate during NMP
