# Supplementary material for: Evaluation of the ex vivo liver viability using a nuclear magnetic resonance relaxation time-based assay in a porcine machine perfusion model
Source: Sci Rep. 2021 Feb 18;11:4117. doi: 10.1038/s41598-021-83202-3 (PMC7892848; doi:10.1038/s41598-021-83202-3)
Supplement: Supplementary file 4 — Supplementary Table S2. [file 41598_2021_83202_MOESM4_ESM.docx]

|  | WIT:0min (n=5) | WIT:30min (n=6) | WIT:60min (n=6) |
| --- | --- | --- | --- |
| Stroma necrosis (grade:0-3) | 0.4(0-1) | 0.6(0-1) | 2(2) |
| Extramural peribiliary glands loss(grade:0-2) | 1(0-2) | 1.5(1-2) | 1.5(1-3) |
| Peribiliary vascular plexus(grade:0-2) | 1(1) | 1.3(1-2) | 2.2(2-3) |

**Supplementary table 2.** Items and degree of bile duct injury in liver grafts of this study accessed via histological scoring
